# Network pharmacology of triptolide in cancer cells: implications for transcription factor binding

**DOI:** 10.1007/s10637-021-01137-y

**Published:** 2021-07-02

**Authors:** Ean-Jeong Seo, Mona Dawood, Annika K. Hult, Martin L. Olsson, Thomas Efferth

**Affiliations:** 1 Department of Pharmaceutical Biology, Institute of Pharmaceutical and Biomedical Sciences, Johannes Gutenberg University, Staudinger Weg 5, 55128 Mainz, Germany; 2 Department of Molecular Biology, Faculty of Medical Laboratory Sciences, Al-Neelain University, Khartoum, Sudan; 3 Division of Hematology and Transfusion Medicine, Department of Laboratory Medicine, Lund University, 221 84 Lund, Sweden

**Keywords:** Microarrays, Natural products, Network pharmacology, Phytochemicals, Precision medicine

## Abstract

*Background* Triptolide is an active natural product, which inhibits cell proliferation, induces cell apoptosis, suppresses tumor metastasis and improves the effect of other therapeutic treatments in several cancer cell lines by affecting multiple molecules and signaling pathways, such as caspases, heat-shock proteins, DNA damage and NF-ĸB. *Purpose *We investigated the effect of triptolide towards NF-ĸB and GATA1. *Methods *We used cell viability assay, compare and cluster analyses of microarray-based mRNA transcriptome-wide expression data, gene promoter binding motif analysis, molecular docking, Ingenuity pathway analysis, NF-ĸB reporter cell assay, and electrophoretic mobility shift assay (EMSA) of GATA1. *Results *Triptolide inhibited the growth of drug-sensitive (CCRF-CEM, U87.MG) and drug-resistant cell lines (CEM/ADR5000, U87.MGΔEGFR). Hierarchical cluster analysis showed six major clusters in dendrogram. The sensitive and resistant cell lines were statistically significant (*p* = 0.65 × 10^–2^) distributed. The binding motifs of NF-κB (Rel) and of GATA1 proteins were significantly enriched in regions of 25 kb upstream promoter of all genes. IPA showed the networks, biological functions, and canonical pathways influencing the activity of triptolide towards tumor cells. Interestingly, upstream analysis for the 40 genes identified by compare analysis revealed ZFPM1 (friend of GATA protein 1) as top transcription regulator. However, we did not observe any effect of triptolide to the binding of GATA1 in vitro. We confirmed that triptolide inhibited NF-κB activity, and it strongly bound to the pharmacophores of IκB kinase β and NF-κB in silico. *Conclusion *Triptolide showed promising inhibitory effect toward NF-κB, making it a potential candidate for targeting NF-κB.

## Introduction

Triptolide, a diterpenoid triepoxide, is predominantly an active natural product isolated from the medicinal plant *Tripterygium wilfordii* Hook F (TWHF) [1]. Triptolide exhibits potent pharmacological activities against inflammation, fibrosis, cancer, viral infection, oxidative stress and osteoporosis [2–4]. Triptolide has a similar structure as steroid hormones, and it showed high binding affinity to a nuclear receptor, human estrogen receptor alpha (ERα) [5]. It selectively inhibits the activity of peroxiredoxin I, which has crucial functions in the development of cancer and inflammation [6]. The XBP1 subunit of the transcription factor TFIH core complex is one of the molecular targets of triptolide, which is important for the inhibitory activity of triptolide to RNA polymerase II-mediated transcription [7]. This feature provides one explanation of the activity of triptolide against several diseases such as inflammation and cancer [8–10].

Triptolide shows proapoptotic and anti-proliferative effects on tumor cell lines in vitro and reduces the tumor size or inhibits tumor growth in vivo. It inhibits cell proliferation, induces cell apoptosis, suppresses tumor metastasis and improves the effect of other therapeutic treatments in several cancer cell lines [11]. It affects multiple molecules and signaling pathways, such as caspases, heat-shock proteins, DNA damage and NF-ĸB. It also enhances chemoradiosensitivity in cancer therapy [12].

Nowadays, targeting transcription factor drivers in cancer is becoming a successful strategy for treatment of cancer [1]. This mainly based on the fact that most of the oncogenes involved in tumorigenesis processes are transcription factors such as forkhead box O (FOXO), nuclear factor kappa B (NF-ĸb), Krüppel-like factor 8 (KLF8), MYC, GATA binding factor (GATA1), activator protein 1 (AP-1), etc. [13, 14].

NF-kB is a family of transcription factors that are constitutively active in different types of tumors. NF-κB has been identified as a key player in carcinogenesis process, since it has a role in activation of cellular pathways such as: cell proliferation, survival, apoptosis, angiogenesis, and metastasis [15]. Hence, targeting NF-κB is of significant therapeutic value. NF-κB is located in the cytoplasm in inactive form by its binding to inhibitory proteins (IκB). In the presence of specific stimuli, IκB is phosphorylated by the IκB kinase (IKK) and eventually degraded. Activated NF-κB is now free to translocate to the nucleus and binds to its specific DNA sequences at the cis-acting elements of the target genes enhancing their expression [16].

The GATA-binding factor (GATA) proteins constitute a large family of transcription factors. In mammals, it is composed of six members (GATA1-GATA6) that can be further classified into two subfamilies based on their expression profile and the structure of the gene [17]. GATA1 and its friend Zinc Finger Protein (ZFPM1, FOG1) are transcription factors and transcription regulators, respectively [18]. They regulate the differentiation of the erythroid and megakaryocytic cell lineages by regulating the expression of the key genes related to cell proliferation, cell differentiation, and apoptosis [19]. ZFPM1 binds to GATA1 to form a heterodimer complex to synergistically activate transcription at the specific regulatory region of the genes. Then, the expressed genes enable differentiation of hematopoietic cells to both erythroid and megakaryocytic cells [20]. Moreover, several studies reported that the deregulation of ZFPM1 and its pathway contributes to the initiation of hematologic malignancies. Therefore, GATA1 is considered a potential target for cancer therapy [21].

In this study, we investigated the cytotoxic activity of triptolide in tumor cell lines. Moreover, we carried out COMPARE and hierarchical cluster analyses for 60 cell lines of the National Cancer Institute (NCI, United States) that represent 9 different types of tumors. Then, we were interested to perform pathway and motif analyses using the 40 genes identified by the microarray. Later, we examined the inhibitory effect of triptolide towards NF-ĸB using in silico molecular docking and NF-ĸB reporter cell assay, since our bioinformatics analysis showed that triptolide affects NF-ĸB. Besides, we also studied the effect of triptolide to GATA proteins, because GATA proteins were predicted to bind triptolide by our motif binding analyses in this study.

## Materials and methods

### Cell lines

Drug-sensitive CCRF-CEM and multidrug-resistant P-glycoprotein-overexpressing CEM/ADR5000 leukemic cells were kindly given by Prof. Axel Sauerbrey (Department of Pediatrics, University of Jena, Germany). Cells were cultured in RPMI1640 medium supplemented with 10% fetal bovine serum (FBS) and 1% penicillin (1,000 U/mL)/streptomycin (100 µg/mL) (P/S) (Life Technologies, Darmstadt, Germany). Doxorubicin (5,000 ng/mL) was supplied to retain overexpression of P-gp (*MDR1, ABCB1*) in resistant CEM/ADR5000 leukemic cells [26]. Human brain glioblastoma cell line U87.MG and the U87.MGΔEGFR that is transfected with a plasmid carrying an *EGFR* gene with a deletion of exons 2–7 were obtained from Dr. W. K. Cavenee (Ludwig Institute for Cancer Research, San Diego, CA, United States). The cell lines were cultured with 800 ng/mL geneticin [22, 23].

The panel of 60 human tumor cell lines of the Development of Therapeutics Program of the National Cancer Institute (NCI, USA) included leukemia, melanoma, non-small cell lung cancer, colon cancer, renal cancer, ovarian cancer, breast cancer, prostate carcinoma cells, and tumor cells of the central nervous system [24].

### Cell viability assay

**Fig. 1:**
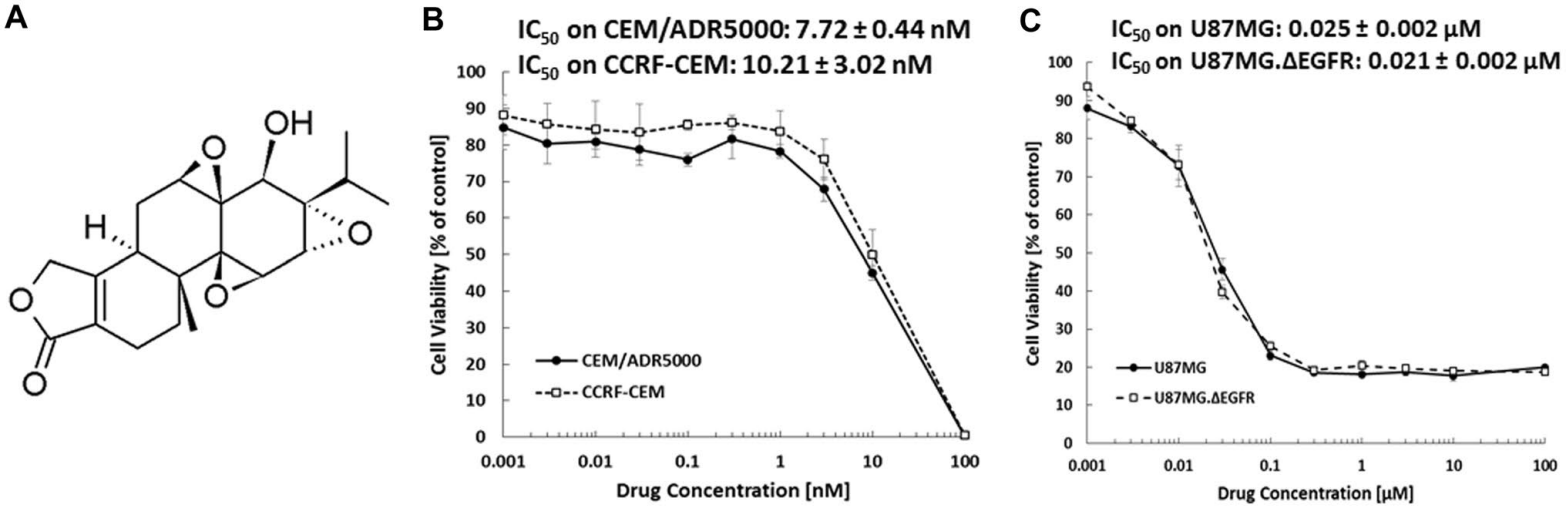
Cytotoxicity of triptolide against cancer cells. Chemical structure of triptolide **A**. Cytotoxic effect of triptolide against CEM/ADR5000 and CCRF-CEM **B**, and U87.MG and U87.MGΔEGFR **C**

The cytotoxic activities of triptolide (Sigma Aldrich, Taufkirchen, Germany; Fig. 1A) were evaluated by the resazurin assay [25]. This assay is based on reduction of the indicator dye, resazurin, to the highly fluorescent resorufin by viable cells. Aliquots of 5,000 cells/100 µL of U87.MG and U87.MGΔEGFR were placed in 96-well plates and incubated for one day before treatment. However, for leukemic cells, 10,000 cells/100 µL cells were seeded into 96-well plates and immediately treated. Twenty microliters of resazurin 0.01% w/v solution were added to each well after 72 h at 37 °C incubation, and the plates were incubated at 37 °C for 4 h. Fluorescence was detected by an Infinite M2000 Proplate reader (Tecan, Crailsheim, Germany) with an excitation wavelength of 544 nm and an emission wavelength of 590 nm. Each experiment was carried out at least three times with six replicates each. The viability was analyzed based on a comparison with untreated cells. Fifty percent inhibition (IC_50_) values imply the drug concentrations needed to inhibit 50% of cell proliferation and were calculated from a calibration curve by linear regression using Microsoft Excel [26, 27].

### Compare and cluster analyses of microarray-based mRNA transcriptome-wide expression data

The mRNA microarray hybridization of the NCI cell lines has been published at the NCI Web site (http://dtp.nci.nih.gov) [28, 29]. COMPARE analyses were used to obtain rank-ordered lists of genes expressed in the NCI cell lines. The detailed method as a tool to determine candidate genes for drug resistance and sensitivity has been previously published [30–33]. In order to identify COMPARE rankings, a scale index of correlation coefficients (R-values) was generated from Iog_10_IC_50_ values of test compounds and microarray-based mRNA expression values. Greater mRNA expression correlated with enhanced drug resistance in the standard COMPARE, whereas greater mRNA expression in cell lines indicated drug sensitivity in reverse COMPARE analyses. Pearson´s correlation test was used to calculate significance values and rank correlation coefficients as relative measure for the linear dependency of two variables.

For hierarchical cluster analyses, objects were classified by calculation of distances according to the closeness of between individual distances. All objects were displayed into cluster trees (dendrograms). Merging of objects with similar features leads to cluster formation, where the length of the branch implies the degree of relation. Distances of subordinate cluster branches to superior cluster branches serve as criteria for the closeness of clusters. Therefore, objects with tightly related features were clustered closely together, if separation of objects in the dendrogram increased with progressive dissimilarity. Hierarchical clustering and heat map analyses were carried out using clustered image map (CIM) miner software by the one matrix CIM (https://discover.nci.nih.gov/cimminer/oneMatrix.d) [34].

### Transcription factor gene promoter binding motif analysis

The top 40 genes, which directly or inversely correlated with log_10_IC_50_ values of the NCI cell lines in COMPARE analysis, were submitted to binding motif analysis. Promoter sequences 25 kb upstream of exon 1 of the corresponding genes were retrieved from UCSC Genome Browser Gene Sorter (http://genome.ucsc.edu). Promoter sequences were checked using the SeqPos tool implemented in the Galaxy Cistrome software [35].

### Molecular docking

The interaction energy of triptolide with NF-ĸB pathway proteins was predicted using molecular docking: I-ĸB kinase β, I-ĸB kinase β-NEMO (NF-ĸB essential modulator) complex, NF-ĸB, and NF-ĸB-DNA complex. The protocol for molecular docking was reported by us [36]. Protein structures using X-ray crystallography were obtained from PDB database (http://www.rcsb.org/). I-ĸB kinase β (PDB ID:3RZF), I-ĸB kinase β-NEMO complex (PDB ID:3BRT), NF-ĸB (p52/RelB heterodimer, PDB ID:3DO7), and NF-ĸB-DNA complex (p50/p65 heterodimer bound to DNA, PDB ID: IVKX) were used in our study.

A grid box was defined for docking spaces in each protein according to its pharmacophores. Docking parameters were set to 250 runs and 2,500,000 energy evaluations for each docking. Dockings were performed three times independently. Lamarckian Genetic Algorithm was chosen for docking calculations. For the visualization of docking results, AutodockTools-1.5.7rcl was used. The surface representation image showing the binding pocket of proteins was made with Visual Molecular Dynamics (VMD) software developed with NIH support by the Theoretical and Computational Biophysics group at the Beckman Institute, University of Illinois at Urbana-Champaign (http://www.ks.uiuc.edu/Research/vmd/).

### Ingenuity pathway analysis

Deregulated genes identified by COMPARE analysis as factors determining cellular responsiveness to triptolide were subjected to pathway analysis, in order to determine the biological function of these genes. Forty genes were imported into the Ingenuity Pathway Analysis (IPA) software (Qiagen Bioinformatics, Redwood City, CA, U.S.A) in Excel format [37].

Core analyses were carried out with general settings: “human” as species and “cell line” as type of biological sample. Canonical pathways, diseases and functions, and upstream regulators were determined by Fisher’s exact test at a significance value of *p* < 0.05. IPA core analyses identifies key regulators and networks in human cell lines [37, 38].

### NF-ĸB reporter cell assay

HEK293 cells stably expressing HEK-Blue-Null1 vector and SEAP on a NF-ĸB promoter were obtained from Invivogen (San Diego, CA, USA). The cells were maintained according to manufacturer´s protocol and treated with various concentrations of triptolide (0 µM, 1.6 µM, 3.1 µM, 6.3 µM, 12.5 µM, 25 µM and 50 µM) for 1 h and triptolide was not removed. Afterwards, NF-ĸB activity was induced with 100 ng/mL of TNF-α for 24 h. The activation was evaluated by detecting SEAP spectrophotometrically at 630 nm with addition of Quanti Blue (Invivogen). The procedure has been published by us [39–41].

### Electrophoretic mobility shift assay (EMSA)

Electrophoretic mobility shift assays were carried out as previously demonstrated [42]. And the sequence of the biotinylated probes (sequence from a regulatory region in *ABO* intron 1 [GenBank KC841429]) used for testing are shown in Table 1. Gel shifts were performed using LightShift™ Chemiluminescent EMSA Kit (Thermo-Fisher, Waltham, MA, USA) and nuclear extracts were prepared from K562 cells (ATCC CCL -243™) [43]. Triptolide was dissolved in DMSO to a 20 mM stock solution which was then further diluted. Nuclear extracts were pre-incubated with three different dilutions of triptolide (5 nM, 10 nM, and 20 nM) for 5 min and three concentrations (2.5 nM, 5 nM, and 100 nM) for 10 min to test if triptolide would inhibit GATA1 protein binding to the probes. The final dilution of DMSO was 0.1%. As a vehicle control, pre-incubation was performed with 0.1% DMSO only and as a negative binding control a probe with a disrupted GATA1 site was used. Supershift assay was performed with polyclonal anti-GATA-1 IgG (1 µg/µL; Active Motif, Waterloo, Belgium).

**Table 1:** EMSA probe designations and sequences (wildtype and mutated GATA1-binding motif highlighted in bold)

Name	Sequence 5’ to 3’
ABOi1 GATA F	AGAGTCTTCGCAATGCCTGGGAAAGGGAGA**GATA**AGGCTCACTAGCCA
ABOi1 GATA R	TGGCTAGTGAGCCTTATCTCTCCCTTTCCCAGGCATTGCGAAGACTCT
ABOi1 GAGA F	AGAGTCTTCGCAATGCCTGGGAAAGGGAGA**GAGA**AGGCTCACTAGCCA
ABOi1 GAGA R	TGGCTAGTGAGCCTTCTCTCTCCCTTTCCCAGGCATTGCGAAGACTCT

## Results

### Cytotoxicity of triptolide towards ABC-transporter expressing tumor cell lines

The cell viability of drug-sensitive (CCRF-CEM, U87.MG) and drug-resistant cell lines (CEM/ADR5000, U87.MGΔEGFR) by triptolide was tested by resazurin assay. Triptolide inhibited the growth of all four cell lines after 72 h. The IC_50_ values of triptolide against CCRF-CEM and CEM/ADR5000 were 10.21 and 7.72 nM, respectively (Fig. 1B). Fifty percentages of U87MG and U87MG.ΔEGFR were inhibited with 0.025 and 0.021 µM triptolide, respectively (Fig. 1C).

### Compare and hierarchical cluster analysis of mRNA microarray data

We studied the transcriptome-wide mRNA expression in 60 NCI cell lines of diverse tumor types using COMPARE analysis and correlated the mRNA expression data with the log_10_IC_50_ values for triptolide, in order to identify novel molecular determinants for this compound. The scale ranking of genes, which were identified by COMPARE analysis, were applied to Pearson´s rank correlation tests. Table 2 shows the top 20 genes with direct and the top 20 genes with inverse correlation coefficients.

**Table 2:** Correlation of constitutive mRNA expression of genes identified by COMPARE analyses with log_10_ IC_50_ values of triptolide for 60 NCI tumor cell lines ^a^

COMPARE coefficient	Pattern ID	Gene Bank accession	Gene abbreviation	Gene name	Gene function
0.480	GC29777	U72514	*EMG1*	EMG1 nucleolar protein homologue (*S. cerevisiae*)	S-adenosyl-L-methionine-dependent pseudo-uridine N(1)-methyltransferase that methylates pseudo-uridine at position 1248 (Psi1248) in 18S rRNA
0.469	GC31760	AI816034	*NHP2*	NHP2 ribonucleoprotein homologue (yeast)	Ribosome biogenesis and telomere maintenance
0.454	GC37113	U18991	*RPE65*	Retinal pigment epithelium-specific protein 65 kDa	Production of 11-cis retinal and in visual pigment regeneration. The soluble form binds vitamin A (all-trans-retinol), making it available for LRAT processing to all-trans-retinyl ester
0.454	GC39388	AF038664	*B4GALT6*	UDP-Gal:βGlcNAc β 1,4- galactosyltransferase, polypeptide 6	Biosynthesis of glycosphingolipids
0.451	GC38177	AB007893	*PPIP5K2*	Diphosphoinositol pentakisphosphate kinase 2	Regulation of apoptosis, vesicle trafficking, cytoskeletal dynamics, exocytosis, insulin signaling and neutrophil activation
0.443	GC39329	D87468	*ARC*	Activity-regulated cytoskeleton-associated protein	Consolidation of synaptic plasticity as well as formation of long-term memory. Required in the stress fiber dynamics and cell migration
0.427	GC27848	AB015633	*TMEM5*	Transmembrane protein 5	Biosynthesis of the phosphorylated O-mannosyl trisaccharide (N-acetylgalactosamine-beta-3-N-acetylglucosamine-beta-4-(phosphate-6-)mannose), a carbohydrate structure present in α-dystroglycan (DAG1), which is required for binding laminin G-like domain-containing extracellular proteins with high affinity
0.426	GC30255	AI972631	*SRRT*	Serrate RNA effector molecule homologue (*Arabidopsis*)	Mediator between the cap-binding complex (CBC) and the primary microRNAs (miRNAs) processing machinery during cell proliferation
0.425	GC37400	D86956	*HSPH1*	Heat shock 105 kDa/110 kDa protein 1	Prevents the aggregation of denatured proteins in cells under severe stress, on which the ATP levels decrease markedly
0.413	GC30551	Y18418	*RUVBL1*	RuvB-like 1 (*E. coli*)	Component of the NuA4 histone acetyltransferase complex which is involved in transcriptional activation of select genes principally by acetylation of nucleosomal histones H4 and H2A
0.413	GC36812	J03626	*UMPS*	Uridine monophosphate synthetase	Uridine monophosphate synthase, pyrimidine de novo biosynthesis
0.410	GC27815	S78798	*PIP4K2A*	Phosphatidylinositol-5-phosphate 4-kinase, type II, α	Catalyzes the phosphorylation of phosphatidylinositol 5-phosphate (PtdIns5P) on the fourth hydroxyl of the myo-inositol ring, to form phosphatidylinositol 4, 5-bisphosphate (PtdIns(4,5)P2)
0.407	GC27991	S43855	*RCVRN*	Recoverin	Calcium-bound recoverin prolongs photoresponse
0.407	GC36672	M94314	*RPL24*	Ribosomal protein L24	60S ribosomal protein L24
0.401	GC28309	Y08991	*PIK3R4*	Phosphoinositide-3-kinase, regulatory subunit 4	Regulatory subunit of the PI3K complex that mediates formation of phosphatidylinositol 3-phosphate
0.395	GC28236	U95032	*GAS2*	Growth arrest-specific 2	Is cleaved during apoptosis and the cleaved form induces dramatic rearrangements of the actin cytoskeleton and potent changes in the shape of the affected cells
0.394	GC35423	X53777	*RPL17*	Ribosomal protein L17	60S ribosomal protein L17
0.393	GC31189	AF081280	*NPM3*	Nucleophosmin/nucleoplasmin 3	Chaperone
0.390	GC30306	U66035	*TIMM8A*	Translocase of inner mitochondrial membrane 8 homologue A (yeast)	Mitochondrial intermembrane chaperone that participates in the import and insertion of some multi-pass transmembrane proteins into the mitochondrial inner membrane
0.388	GC35656	D79995	*TTLL4*	Tubulin tyrosine ligase-like family, member 4	Polyglutamylase which preferentially modifies beta-tubulin and nucleosome assembly proteins NAP1 and NAP2
-0.581	GC26920	AB029035	*ARHGEF4*	Rho guanine nucleotide exchange factor (GEF) 4	Guanine nucleotide exchange factor (GEF) for RHOA, RAC1 and CDC42 GTPases
-0.571	GC31233	X54131	*PTPRB*	Protein tyrosine phosphatase, receptor type, B	Blood vessel remodeling and angiogenesis
-0.561	GC35073	AB017915	*CHST3*	Carbohydrate (chondroitin 6) sulfotransferase 3	Sulfotransferase that utilizes 3-phospho-5-adenylyl sulfate (PAPS) as sulfonate donor to catalyze the transfer of sulfate to position 6 of the N-acetylgalactosamine (GalNAc) residue of chondroitin
-0.524	GC28183	X54131	*PTPRB*	Protein tyrosine phosphatase, receptor type, B	Blood vessel remodeling and angiogenesis
-0.515	GC28807	Y09836	*MAP1B*	Microtubule-associated protein 1B	Facilitates tyrosination of α-tubulin in neuronal microtubules
-0.494	GC33702	D64053	*PTPRR*	Protein tyrosine phosphatase, receptor type, R	Sequesters mitogen-activated protein kinases (MAPKs) such as MAPK1, MAPK3 and MAPK14 in the cytoplasm in an inactive form
-0.492	GC30612	S40719	*GFAP*	Glial fibrillary acidic protein	A class-III intermediate filament. It is a cell-specific marker that, during the development of the central nervous system
-0.487	GC39169	D78014	*DPYSL3*	Dihydropyrimidinase-like 3	Necessary for signaling by class 3 semaphorins and subsequent remodeling of the cytoskeleton
-0.486	GC33701	D64053	*PTPRR*	Protein tyrosine phosphatase, receptor type, R	Sequesters mitogen-activated protein kinases (MAPKs) such as MAPK1, MAPK3 and MAPK14 in the cytoplasm in an inactive form
-0.475	GC31046	X75342	*SHB*	Src homology 2 domain containing adaptor protein B	Adapter protein which regulates several signal transduction cascades by linking activated receptors to downstream signaling components
-0.468	GC30046	X65724	*NDP*	Norrie disease (pseudo-glioma)	Activates the canonical Wnt signaling pathway through FZD4 and LRP5 coreceptor
-0.465	GC30818	U49082	*SLC38A3*	Solute carrier family 38, member 3	Sodium-dependent amino acid/proton antiporter. Mediates electrogenic cotransport of glutamine and sodium ions in exchange for protons
-0.461	GC29160	AF011375	*ITGB4*	Integrin, β 4	Integrin α-6/ β-4 is a receptor for laminin. Structural role in the hemidesmosome of epithelial cells. Regulation of keratinocyte polarity and motility
-0.459	GC35360	M26252	*PKM*	Pyruvate kinase, muscle	Glycolytic enzyme that catalyzes the transfer of a phosphoryl group from phosphoenolpyruvate (PEP) to ADP, generating ATP
-0.455	GC32168	Z35307	*ECE1*	Endothelin converting enzyme 1	Converts big endothelin-1 to endothelin-1
-0.451	GC35300	X63432	*ACTB*	Β-Actin	Involved in cell motility. Ubiquitously expressed in all eukaryotic cells
-0.449	GC38666	AF002715	*MAP3K4*	Mitogen-activated protein kinase kinase kinase 4	Component of a protein kinase signal transduction cascade. Activates the CSBP2, P38 and JNK MAPK pathways. Specifically phosphorylates and activates MAP2K4 and MAP2K6
-0.448	GC33904	MAP2K1	*L05624*	Mitogen-activated protein kinase kinase 1	Dual specificity protein kinase which acts as an essential component of the MAP kinase signal transduction pathway. Binding of extracellular ligands such as growth factors, cytokines and hormones to their cell-surface receptors activates RAS and this initiates RAF1 activation
-0.447	GC38065	X97868	*ARSF*	Arylsulfatase F	Arylsulfatase F, which is not inhibited by DHEAS or warfarin
-0.441	GC34659	M62896	*ANXA2P1*	Annexin A2 pseudogene 1	Annexin A2, pseudogene 1

^a^ Positive correlation coefficients indicate direct correlations to log_10_ IC_50_ values; negative ones indicate inverse correlations. Information on gene functions was taken from the OMIM database, NCI, USA (http://www.ncbi.nlm.nih.gov/Omim/) and from the GeneCard database of the Weitzman Institute of Science, Rehovot, Israel (http://bioinfo.weizmann.a.il/cards/index.html)

**Fig. 2:**
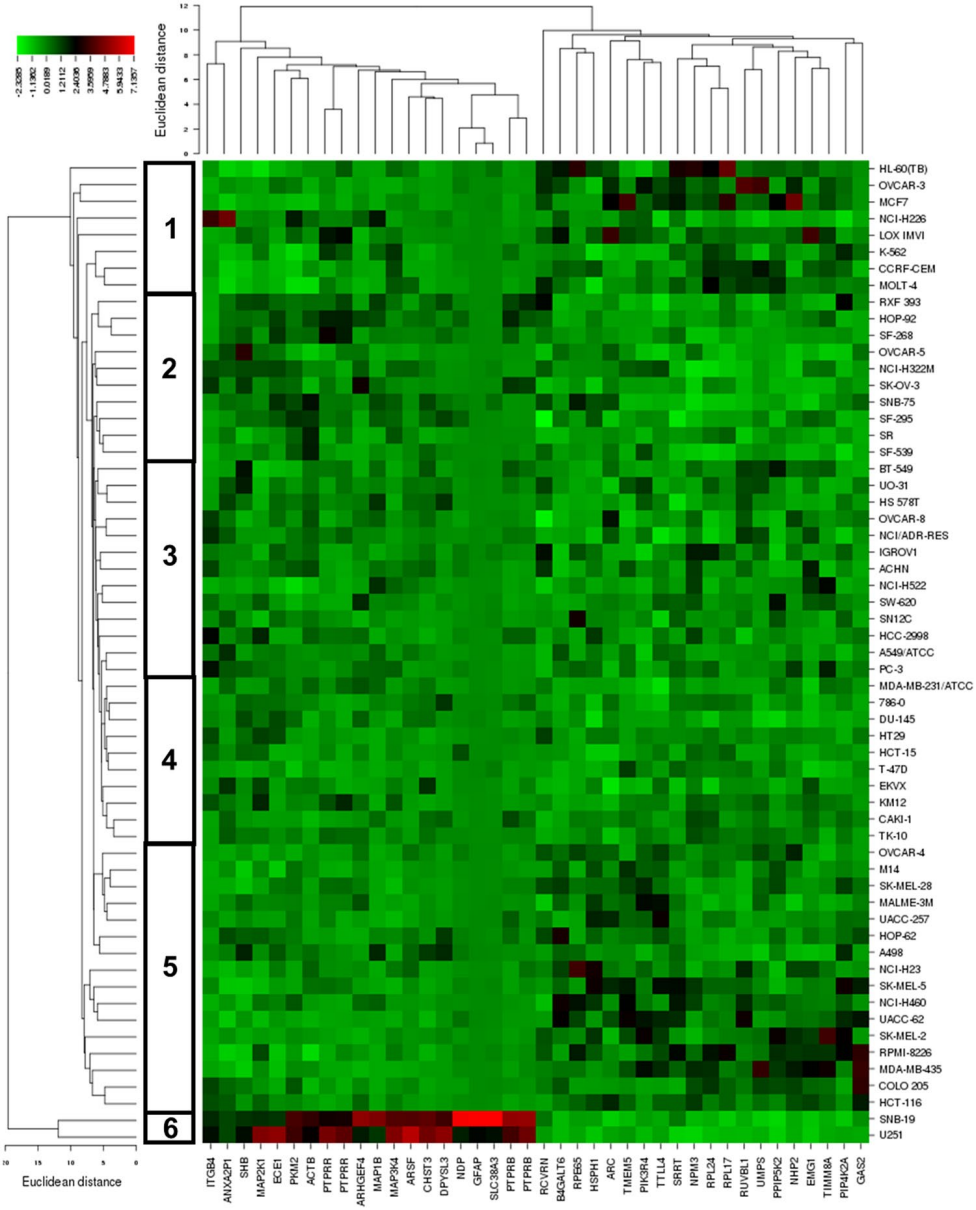
Hierarchical cluster analysis and heatmap of genes expressions involved in cancer cell sensitivity to triptolide

Hierarchical cluster analysis and cluster image mapping were performed with the mRNA expression of these genes (Fig. 2). The dendrogram of the heat map can be separated into six major clusters (Fig. 2). Cluster 1 and 5 included mainly sensitive, cluster 2, 3 and 4 contained mostly resistant cell lines. Cluster 6 has only resistant cell lines. The distribution of sensitive and resistant cell lines showed statistically significant (*p* = 0.65 × 10^–2^) (Table 3).

**Table 3:** Separation of clusters of NCI cell lines obtained by hierarchical cluster analyses for triptolide shown in Fig. 2

	Sensitive	Resistant
Partition	≤ -7.948	> -7.948
Cluster 1	6	2
Cluster 2	3	7
Cluster 3	5	8
Cluster 4	2	8
Cluster 5	13	3
Cluster 6	0	2
Chi-square test	*p* = 0.65 × 10^–2^	

### Transcription factor binding motif analysis in gene promoters

**Fig. 3:**
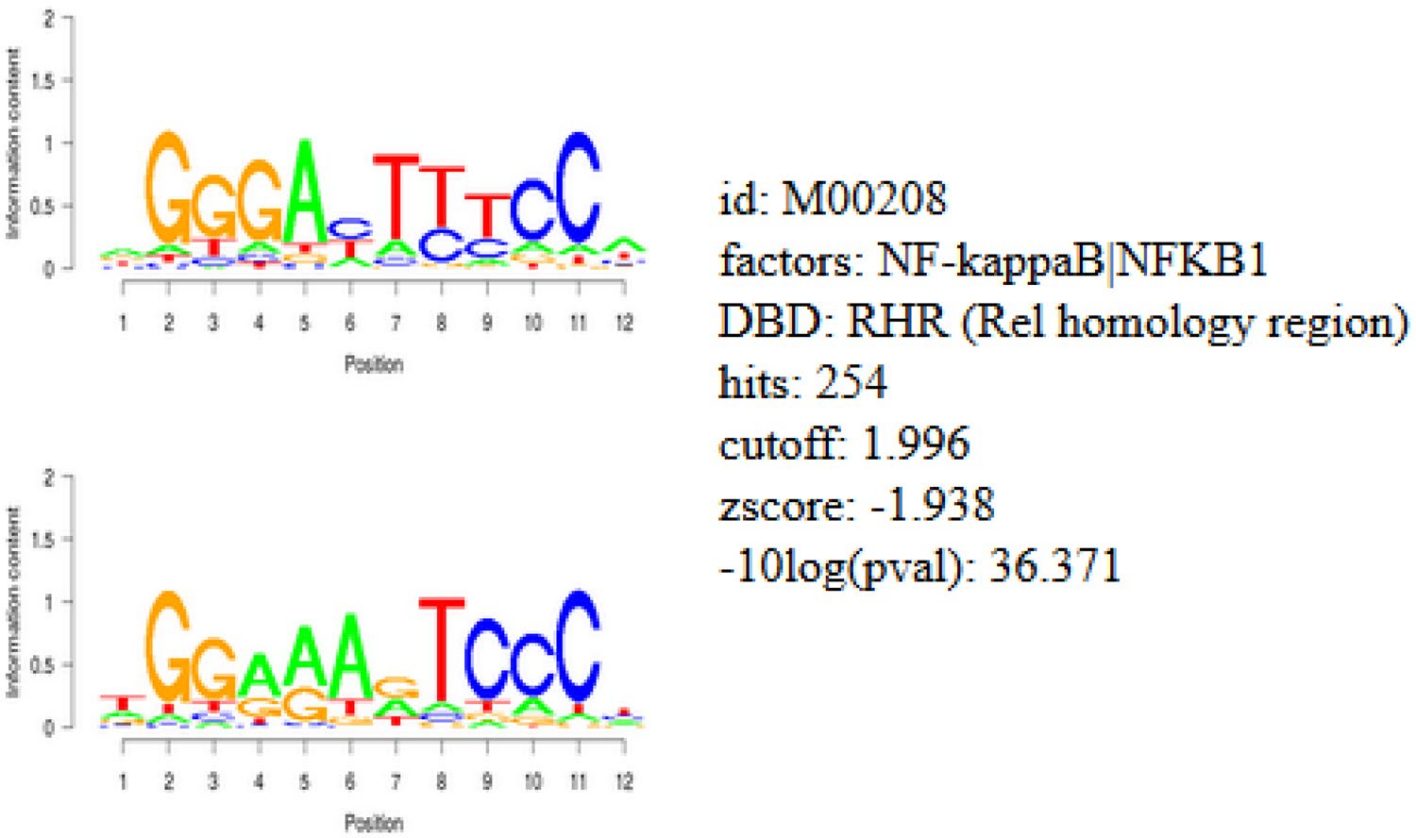
Binding motif analysis of 25 kb upstream regions of 40 genes identified by COMPARE analysis revealing the significant presence of NF-κB binding motifs

**Fig. 4:**
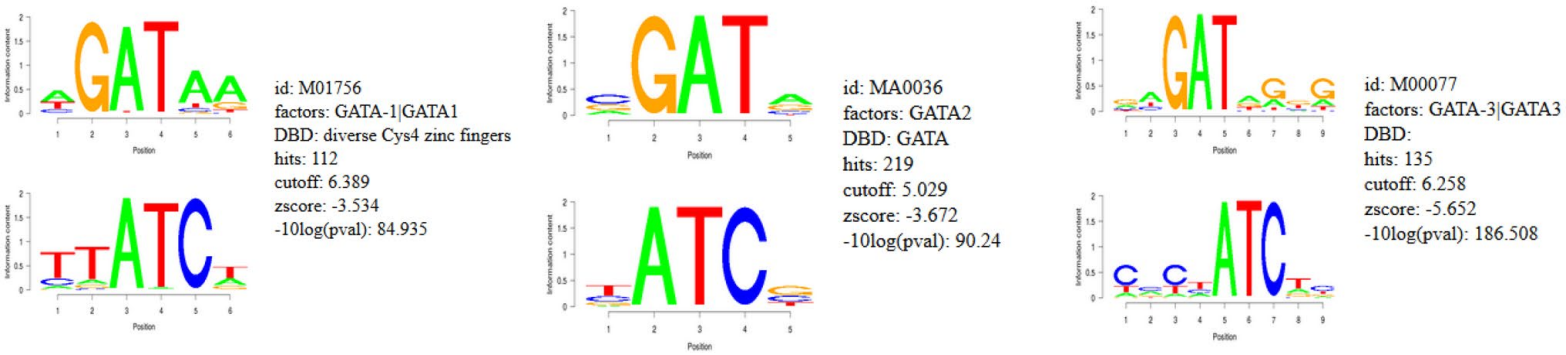
Binding motif analysis of 25 kb upstream regions of 40 genes identified by COMPARE analysis revealing the significant presence of GATA proteins binding motif

Forty genes were identified by COMPARE analysis and the promoters of these genes contained transcription factor binding motifs for NF-κB and GATA proteins (GATA1, 2 and 3) (Figs. 3 and 4). The NF-κB DNA binding motif (Rel) was significantly enriched (with a log *p*-value of -3.6) in regions of 25 kb upstream promoter of all genes, with 254 hits and a Z-score of -1.94 (Fig. 3). This analysis demonstrated that NF-κB plays a crucial role in the regulation of genes related to triptolide, as it was reported before [11, 12]. Furthermore, the binding motifs of GATA proteins 1, 2 and 3 were widely distributed in the promoter regions of all genes, with 112 hits, a Z-score of -3.5, with 219 hits, a Z-score of -3.7 and with 135 hits, a Z-score of -5.7, respectively (Fig. 4).

### Molecular docking

In order to study the interaction of triptolide with the NF-κB pathway in more detail, molecular docking analyses were carried out using IκB kinase β, IκB kinase β-NEMO, NF-κB, NF-κB DNA complex in silico. Triptolide strongly bound to the pharmacophores of IκB kinase β and NF-κB DNA complex. Triptolide bound to IκB kinase β with a binding energy of -7.85 kcal/mol and to NF-κB DNA complex with a binding energy of -7.68 kcal/mol (Table 4).

**Table 4:** In silico molecular docking of triptolide on NF-ĸB Pathway proteins. Dockings were performed with 250 runs for each protein

Protein	Lowest energy of docking (kcal/mol)	Mean binding energy (kcal/mol)	Residues involved hydrogen bond interaction with the ligand	Residues involved in hydrophobic interaction with ligand	pKi (nM)
IĸB kinase β	-7.85 ± 0.26	-7.78 ± 0.32	LYS44, GLU100	LEU21, THR23, VAL29, ALA42, LYS44, VAL74, MET96, TYR98, CYS99, GLU100, GLY102, GLU149, ASN150, VAL152, ILE165, ASP166, LEU167	1.89 ± 0.91
IĸB kinase β- NEMO	-6.16 ± 0.02	-6.08 ± 0.01	-	MET94, PHE97, ALA100, ARG101, VAL104	30.72 ± 0.12
NF-ĸB	-5.88 ± 0.12	-5.86 ± 0.13	PHE273, PHE276, ARG290	GLN271, ALA272, PHE273, GLY274, ASP275, PHE276, ARG290, PRO292	49.70 ± 10.61
NF-ĸB DNA complex	-7.68 ± < 0.00	-7.66 ± < 0.00	DA18	DT8, DT9, DA18, DG19, DT20, ARG124	2.35 ± < 0.00

### Ingenuity pathway analysis

**Fig. 5:**
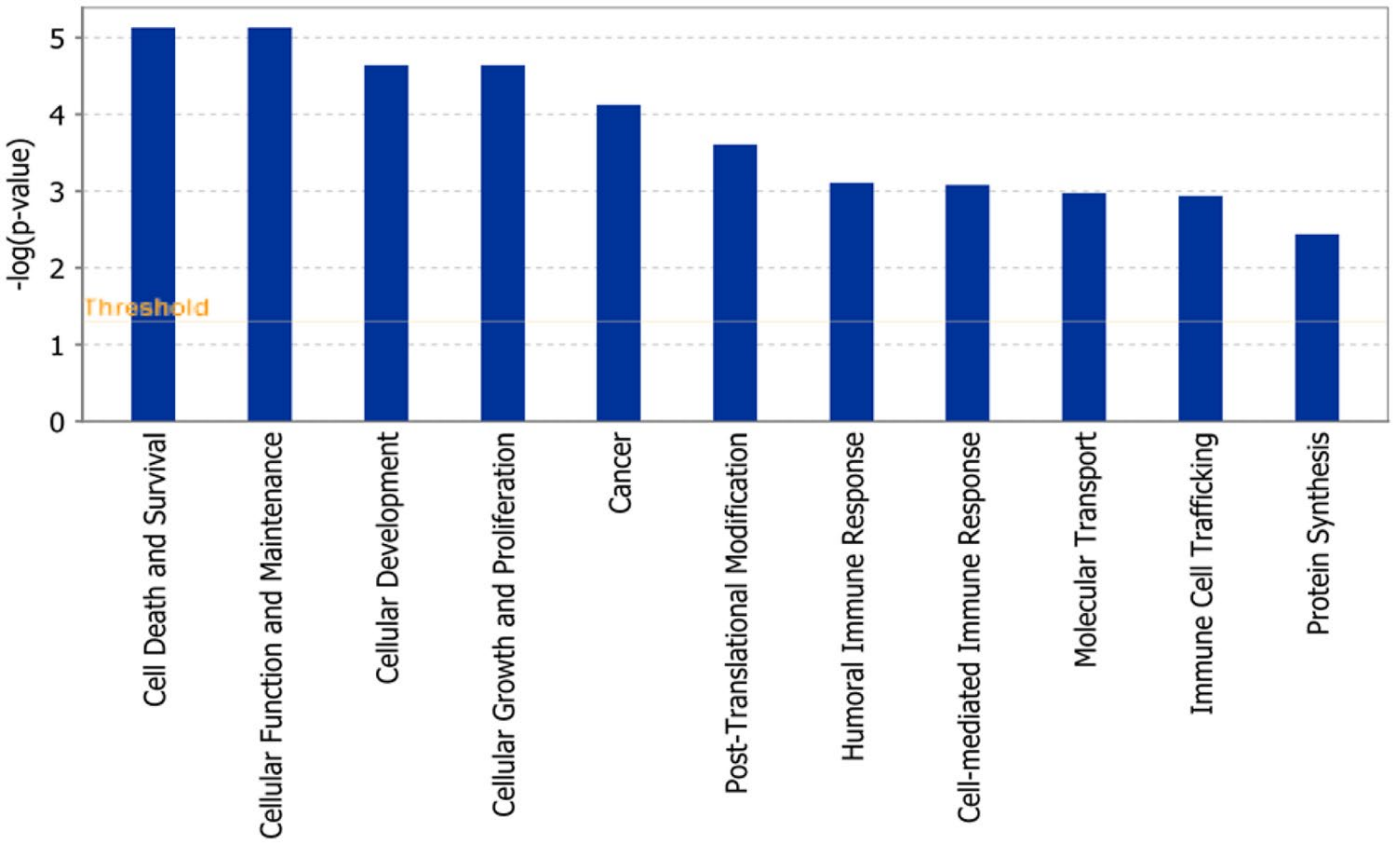
Biological functions affected by triptolide as determined by mRNA microarray hybridization and ingenuity pathway analysis

Deregulated genes identified by COMPARE analysis were subjected to IPA. Interestingly, pathways regulating cell death and survival, cellular development, cellular growth, cancer, post translation modification and humoral immune response, etc. appeared as top affected cellular functions and diseases (Fig. 5).

**Fig. 6:**
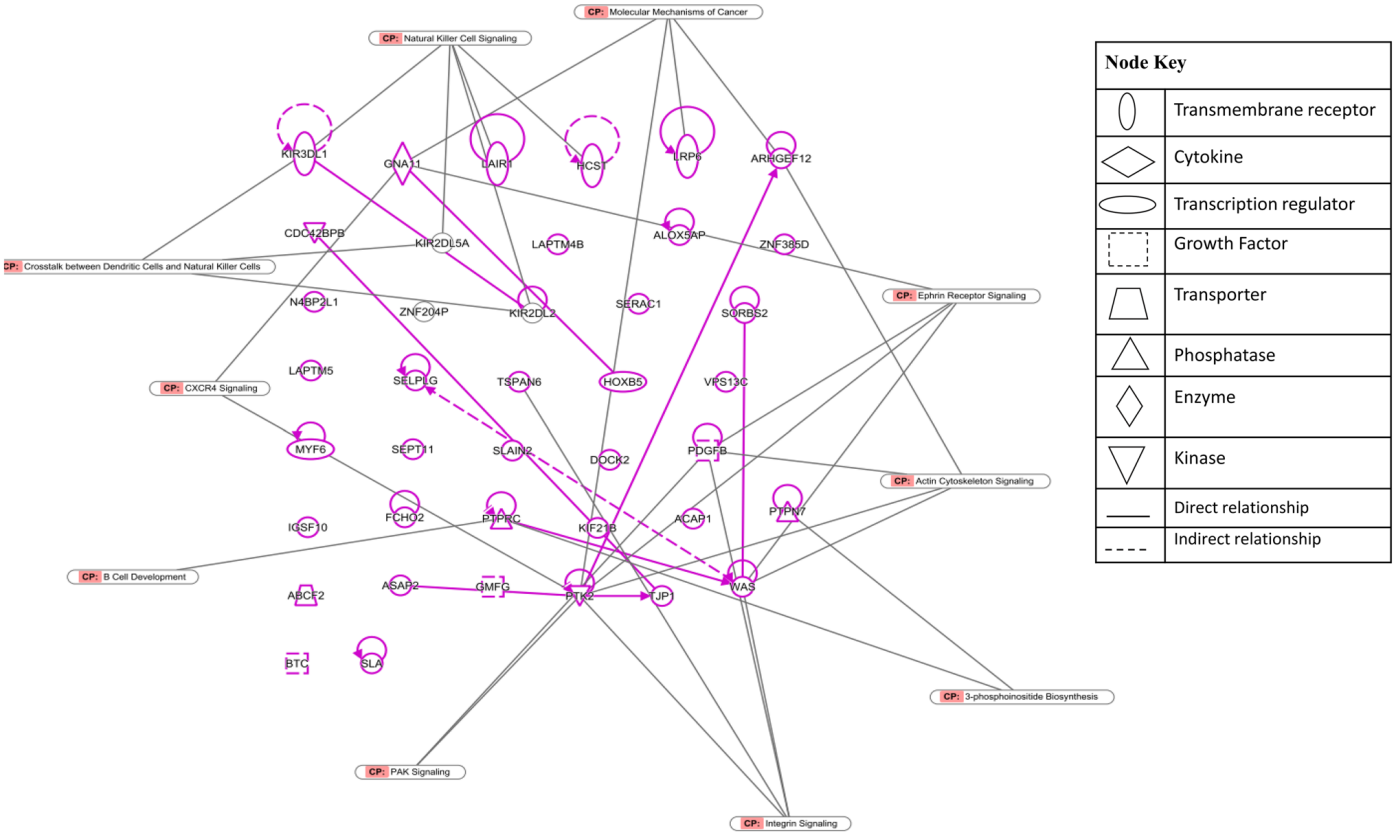
Biological functions affected by triptolide as determined by mRNA microarray hybridization and Ingenuity Pathway Analysis

The canonical pathways analysis revealed that triptolide affects numerous pathways, such as natural killer cell signaling, ephrin receptor signaling, crosstalk between dendritic cells and natural killer cells, integrin signaling, actin cytoskeleton signaling, etc. (Fig. 6). On the other hand, the upstream regulators that were identified using IPA showed ZFPM1 as the top transcription regulators with a *p*-value of 2.85E-04.

### Experimental verification of binding motif analyses

#### NF-ĸB reporter assay

**Fig. 7:**
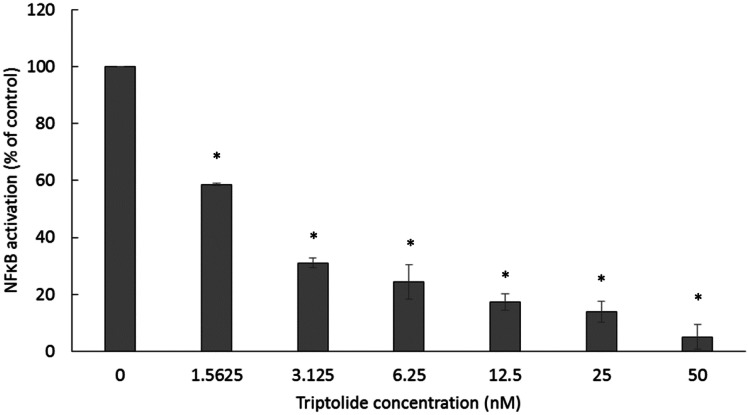
Effect of triptolide on NF-κB activity

Since molecular dockings and motif analysis showed the high binding affinities of triptolide to NF-κB and its regulator, IκB, we carried out NF-κB reporter assay using a SEAP-driven cell line. Triptolide inhibited NF-κB activity in a dose-dependent manner (Fig. 7).

### EMSA of GATA following triptolide incubation

**Fig. 8:**
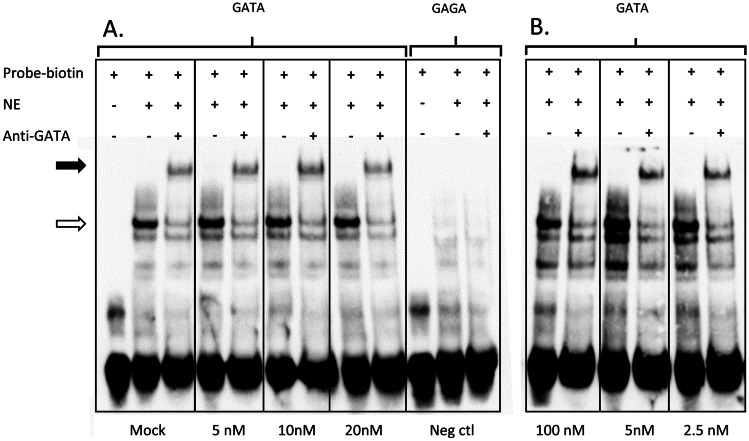
**EMSA** Testing was performed with 48-bp biotinylated probes spanning the GATA1 site investigated. **A** The wild-type probe (GATA) exhibited a shift (white arrow) upon incubation with nuclear extract (NE) from K562 cells, mock-treated or pre-incubated with different concentrations of triptolide (5, 10, and 20 nM), and a further supershift (black arrow) with addition of anti-GATA1, detecting binding of GATA1 to the wild-type probe. As a negative control the probe with a disrupted GATA1 site (GAGA) showed no shift with nuclear extract and hence no supershift could be detected. **B** Further testing was performed (controls not shown) with higher and lower concentrations of triptolide (100 and 2.5 nM) when the incubation time was prolonged from 5 min to 10. For all concentrations tested there are clear shifts and supershifts further indicating that triptolide does not inhibit GATA1 protein binding to the probes

EMSA testing was performed with probes spanning a well-characterized GATA1 site to evaluate if triptolide can inhibit binding of transcription factor GATA1. Pre-incubation of GATA1-containing nuclear extracts with various concentrations of triptolide did not influence the binding pattern of the GATA1 protein to the probes. GATA1 protein binding was verified by addition of anti-GATA1 giving rise to a clear supershift in all cases except for the negative control, for which neither a shift nor a supershift was observed when a probe with a disrupted GATA site was used (Fig. 8).

## Discussion

Triptolide inhibits the proliferation and induces apoptotic cell death in several cancers. Triptolide increases the nuclear accumulation of p53 and apoptotic cell death in human prostatic epithelial cells [44].

Multi-drug resistance (MDR) is an obstacle for cancer therapy [45]. Triptolide inhibited the expression of MDR protein and promoted apoptotic cell death of drug-sensitive parental KB cells and multidrug-resistant KB-7D and KB-tax cells [46]. Besides, triptolide changed the activity of P-glycoprotein drug efflux and mRNA expression of MDR genes [9]. We have comparable results that triptolide strongly inhibited the growth not only of drug-sensitive CCRF-CEM cells but also MDR P-glycoprotein overexpressing CEM/ADR5000 cells in the nanomolar range.

Collateral sensitivity (hypersensitivity) is occasionally observed in ABC-transporter-expressing cells. This event has been well known for several years in ABCB1 (P-gp/MDR1) and MRP1 overexpressing tumor cells [47, 48]. Interestingly, P-glycoprotein overexpressing CEM/ADR5000 cells revealed collateral sensitivity to triptolide in comparison with their parental drug-sensitive counterparts. Collateral sensitivity represents an interesting phenomenon from the therapeutic point of view, because it opens the perspective that drug-resistant refractory tumors could be successfully treated with this kind of drugs [49].

EGFR is a member of the ErbB family of receptors. Upon binding with its ligands, such as EGF or TGF-α, EGFR homo- or hetero-dimerizes with other ErbB family members to activate downstream signaling cascades after tyrosine phosphorylation. This signaling controls several cellular processes, such as proliferation, survival, and apoptosis. EGFR mutations leading to EGFR overexpression result in cancer development, and EGFR mutations affect the poor prognosis of patients and mediate drug resistance of tumors [50]. The in-frame deletion of the extracellular EGFR domain causes ligand-independent receptor activation and represents a common mutant type in brain tumors, termed ΔEGFR [51]. Triptolide showed strong inhibitory effects towards both wtEGFR (sensitive) and ΔEGFR (resistant) with the same concentrations. These results demonstrate that ΔEGFR does not confer resistance against triptolide, indicating that triptolide might be a suitable candidate to treat drug-resistant tumors with ΔEGFR mutation.

The relationship between the gene expression patterns and the drug responses was investigated using the tumor cell line panel of the NCI developmental therapeutic program. We identified a gene expression profile, which was significantly related to the log_10_IC_50_ values of the cell lines to triptolide. COMPARE analysis identified a set of genes from several functional groups (*e.g.,* cell morphology, transmembrane, ribosomal proteins, protein tyrosine phosphatase, and microtubule formation).

Cluster analyses were carried out to predict, whether the cancer cell lines were sensitive or resistant to a cytotoxic drug [52]. The distribution of triptolide-sensitive or -resistant cell lines differed significantly among the different clusters. The portion of sensitive cells in clusters 1 and 5 was much higher than in clusters 2, 3, 4 and 6. Cluster 1 included 75% sensitive cells and 25% resistant cells. Cluster 2 contained 30% sensitive cell lines and 70% resistant cell lines. Cluster 3 contained 38% sensitive cell lines and 62% resistant cell lines. Cluster 4 had 20% sensitive cells and 80% resistant cells. Cluster 5 contained 81% sensitive cell lines and 19% resistant cell lines. Cluster 6 included 100% resistant cell lines. This distribution showed significant differences among the clusters as demonstrated by the chi square test (*p* = 0.65 × 10^–2^). The cluster analysis identified two clusters with predominantly triptolide-sensitive and four clusters with predominantly triptolide-resistant cell lines. What does this mean for cancer therapy? By applying this two-step approach with COMPARE and cluster analyses, it was possible to predict cellular drug response by gene expression profiling of cell lines. It is not beyond the scope of imagination that similar approaches may be applied to predict the sensitivity of individual tumors of patients towards standard chemotherapeutic drugs and also cytotoxic compounds such as triptolide. In case resistance to standard chemotherapy occurs, the oncologist could switch to cytotoxic natural products which are still active in these otherwise resistant tumors. Hence, the estimation of sensitivity or resistance to cytotoxic drugs by mRNA expression profiles may be applied for novel strategies of individualized cancer treatment and precision medicine, because an approach like this could provide opportunity to determine prior to therapy, whether or not an individual tumor would react to specific drugs or natural products.

In addition to the prediction of sensitivity or resistance to specific anticancer compounds, gene expression profiling coupled with COMPARE and cluster analyses can be used to identify relevant molecular mechanisms of triptolide (and other anticancer drugs and natural products) to unravel underlying molecular modes of action of drugs. In the case of triptolide, we found that NF-κB plays an important role for the anticancer activity of triptolide. NF-κB is a ubiquitous transcription factor that controls the expression of genes involved in inflammation, the immune response, cell proliferation, and apoptosis [53]. Interestingly, the NF-κB binding motif was identified in the upstream promoter regions of all genes, which were identified by COMPARE analysis, demonstrating that NF-κB is a crucial transcription regulator of triptolide´s activity in cancer cells.

NF-ĸB is cell nuclear factor, which is related to transcription regulation in the process of inflammation, stress, cell growth and proliferation. NF-ĸB promotes cell proliferation, cell apoptosis and plays a crucial role for the tumor development. NF-ĸB is a heterologous dimer composed of p50 and p65. Triptolide inhibits the transactivation effect of the p65 subunit of NF-ĸB and promotes cell apoptosis [10]. Triptolide also indirectly inhibits NF-ĸB signaling through the AKT/GSK3β/mTOR pathway and induces apoptosis in ovarian cancer by inhibition of NF-ĸB expression [54, 55].

IPA was carried out to predict molecular mechanism of triptolide using COMPARE and hierarchical cluster analyses genes. IPA indeed showed cellular processes revealing the anti-tumor activity of triptolide. Furthermore, it presented the ZFPM1 (friend of GATA1) as a top candidate in the upstream regulators list. These results are in accordance with the data obtained from the motif analyses. Therefore, we wanted to investigate the inhibition of GATA1 function by triptolide. To the best of our knowledge, the effect of triptolide towards GATA1 is described here for the first time. EMSA experiments displayed that triptolide has no apparent effect on GATA1 binding to its motif. One study showed that GATA1 was up-regulated in Sertoli cells upon treatment with triptolide for 15 days. Then, the authors concluded that triptolide has no effect at the level of *Gata1* and the change in the expression was probably due to cellular changes leading to spermatids formation [56].

In conclusion, triptolide showed remarkable cytotoxic effect in different sensitive and drug-resistant cancer cell lines. In particular, P-glycoprotein overexpressing CEM/ADR5000 cells were collateral sensitive toward triptolide. Other investigations were performed to understand the mechanism of action of triptolide. Bioinformatics tools predicted the sensitivity or resistance of tumor cells to triptolide using 60 NCI cell lines. Ingenuity Pathway Analysis identified cellular processes and signaling pathways of genes involved in the mechanisms of action of PT. Finally, triptolide strongly inhibited the activity of NF-κB, while it did not show significant effect towards GATA1.

## Data Availability

All data generated or analysed during this study are included in this published article.
